# Green room temperature synthesis of silver–gold alloy nanoparticles†

**DOI:** 10.1039/d2na00793b

**Published:** 2023-02-13

**Authors:** N. E. Traoré, M. J. Uttinger, P. Cardenas Lopez, D. Drobek, L. Gromotka, J. Schmidt, J. Walter, B. Apeleo Zubiri, E. Spiecker, W. Peukert

**Affiliations:** a Institute of Particle Technology, Friedrich-Alexander-Universität Erlangen-Nürnberg Cauerstraße 4 91058 Erlangen Germany wolfgang.peukert@fau.de; b Interdisciplinary Center for Functional Particle Systems, Friedrich-Alexander-Universität Erlangen-Nürnberg Haberstraße 9a 91058 Erlangen Germany; c Institute of Micro- and Nanostructure Research (IMN), Center for Nanoanalysis and Electron Microscopy (CENEM), Interdisciplinary Center for Nanostructured Films (IZNF), Friedrich-Alexander-Universität Erlangen-Nürnberg Cauerstraße 3 91058 Erlangen Germany

## Abstract

Metallic alloy nanoparticles (NPs) exhibit interesting optical, electrical and catalytic properties, dependent on their size, shape and composition. In particular, silver–gold alloy NPs are widely applied as model systems to better understand the syntheses and formation (kinetics) of alloy NPs, as the two elements are fully miscible. Our study targets product design *via* environmentally friendly synthesis conditions. We use dextran as the reducing and stabilizing agent for the synthesis of homogeneous silver–gold alloy NPs at room temperature. Our approach is a one-pot, low temperature, reaction-controlled, green and scalable synthesis route of well-controlled composition and narrow particle size distribution. The composition over a broad range of molar gold contents is confirmed by scanning transmission electron microscopy-energy-dispersive X-ray spectroscopy (STEM-EDX) measurements and auxiliary inductively coupled plasma-optical emission spectroscopy measurements (ICP-OES). The distributions of the resulting particles in size and composition are obtained from multi-wavelength analytical ultracentrifugation using the optical back coupling method and further confirmed by high-pressure liquid chromatography. Finally, we provide insight into the reaction kinetics during the synthesis, discuss the reaction mechanism and demonstrate possibilities for scale-up by a factor of more than 250 by increasing the reactor volume and NP concentration.

## Abstract 2

Silver–gold alloy nanoparticles of well-controlled composition and narrow particle size distribution *via* a room temperature, reaction-controlled, green and scalable synthesis route.

## Graphical abstract

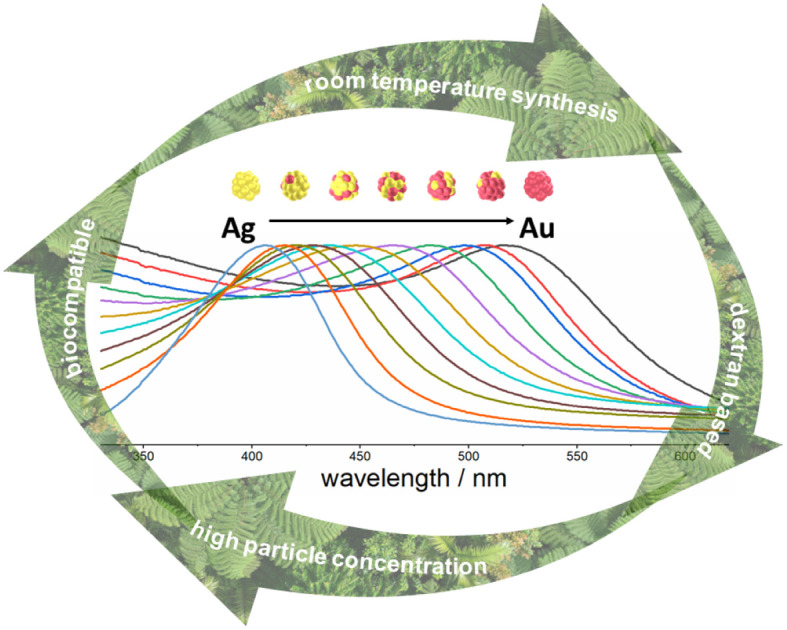

## 1 Introduction

Particles with a nominal size below 100 nm are widely classified as nanoparticles (NPs).^1^ Their size, surface, structure and chemical composition directly define their physical properties, *i.e.* specifically their optical and electrical properties. In recent times, a wide range of NPs have been successfully used in many fields of application such as microelectronics,^2,3^ medicine^4^ and the chemical industry.^5^ In this context, targeted product design strategies of any type of NPs require an iterative approach and multiple optimization steps.^6^ In particular, the NPs' composition, size and shape^7,8^ have to be precisely controlled both in the lab and during scale-up. Therefore, methods to determine those quantities are imperative for the development of promising synthesis routes.

In the context of product design, particulate systems have attracted increasing interest due to their unique functionalities.^9^ Specific examples include quantum dots,^10,11^ which are used in modern displays,^12,13^ solar cells^14,15^ or nanosensors,^16^ and precious metal NPs, which play an important role in plasmonic sensors^17,18^ or catalysis.^19^ Moreover, as many applications depend on the product quality, especially in terms of particle size distribution (PSD) and composition, the development of scalable synthesis strategies for well-controlled particles with a defined composition is mandatory.^20,21^ Examples are quantum dots, whose optical properties are determined by their PSD due to their size-dependent bandgap energy,^22–24^ or gold NPs, whose size influences their distribution in the body and blood circulation times.^25–27^ The latter are especially relevant when used as contrast agents for computed tomography.^27–30^ Alloy NPs find applications in a variety of fields including heterogeneous catalysis.^19,31^ Further, medical applications of alloy NPs show promising results, *e.g.* in the case of hyperthermia treatment of tumors.^4,32^ The anti-bacterial properties of silver NPs^33^ can be combined with the biological inertness of gold NPs in silver–gold alloy NPs, effectively varying the dose of the antibacterial component.^34^ Aside from their specific applications, silver and gold NPs are often used as model alloy systems to better understand the formation of other alloy NPs as silver and gold atoms are fully miscible due to their almost identical lattice constants.^35–37^

A variety of different synthesis routes has been reported in literature. These include gas phase syntheses like flame spray pyrolysis,^38^ as well as more commonly used liquid phase synthesis routes like laser syntheses,^36,37,39,40^ microwave assisted synthesis routes,^41^ electrochemical reduction,^35^ chemical co-reduction^42–45^ and biosynthesis utilizing bacteria^46^ or fungi.^47–49^ The exploited particle formation mechanisms depend strongly on the built-up supersaturation.^37,39,40^ Chemical co-reduction is the most commonly applied route for the synthesis of silver and gold NPs. Co-reduction can be achieved *via* electrochemical routes in a multi-potentiostat,^35^ chemical routes by addition of a reducing agent like sodium borohydride^45^ or biochemical routes by exposure to bacteria^46^ or fungi.^47–49^ It consists of three main steps: (1) the reduction of precursor ions and supersaturation build-up, (2) the nucleation of the free gold and silver atoms to stable nuclei and (3) the growth of nuclei to NPs.^50–55^ The final particles are subsequently stabilized. Commonly, AgNO_3_ is chosen as the silver precursor salt and HAuCl_4_·3H_2_O as the gold precursor salt. Due to the growing need of environmentally friendly methods in the production of NPs, especially green synthesis routes receive increased attention in the field.^56^

While many synthesis routes reported in literature show promising results in terms of the produced NPs' properties, a compromise between harsh process conditions in terms of temperature, energy input and hazardous chemicals on the one hand, and the precise adjustment of the composition of the resulting particles on the other hand, is necessary. For the transfer of lab protocols to industrial production at a larger scale, all these aspects must be considered during scale-up, while keeping all disperse properties, in particular size and composition, constant. The laser, electrochemical and flame spray synthesis routes reported above require dedicated equipment, biochemical reductions with bacteria and fungi require a prior cultivation step.^47,49^ Additionally, the produced NPs usually show a comparably wide or even bimodal PSD,^46–49^ non-spherical shapes^47^ or varying compositions.^46,49^ Moreover, multiple syntheses were reported using citrate at temperatures above 100 °C under vigorous mixing.^42,44^ Few syntheses at room temperature were reported using sodium borohydride and other harmful chemicals.^44,45^ A two-step green synthesis route using dextran as the reducing and capping agent was reported by Bankura *et al.* In this work, silver NPs were produced in the first step and alloys were synthesized by mixing with gold precursor.^57^ The produced alloy NPs, however, were neither tuned to precise molar compositions nor analyzed comprehensively to judge their quality.

In our study, we synthesized silver–gold alloy NPs with accurately adjustable molar compositions and constant mean particle sizes at room temperature using dextran as the reducing as well as the capping agent. The NPs are produced *via* a one-pot, low temperature, reaction-controlled, green and scalable synthesis route. The produced NPs are analyzed with regards to their distribution in size and chemical composition using multi-wavelength analytical ultracentrifugation (MWL-AUC). The hereby generated unique 2D distributions present an excellent measure for the quality of the produced particles with outstanding accuracy, reproducibility and statistical significance. The particles are further characterized by UV-vis spectroscopy, size exclusion chromatography and scanning transmission electron microscopy (STEM). We show how our novel green synthesis route at room temperature results in narrowly distributed alloy NPs with respect to size and composition. The optical properties of the alloy NPs can be directly controlled by varying the alloy composition. Moreover, the alloy NPs are biocompatible due to the use of dextran as a stabilizer, while maintaining green process conditions at atmospheric pressure and room temperature without the need of fast mixing throughout the reaction. The latter aspect largely simplifies scale-up since mass transfer issues during mixing are avoided when moving from small to larger reactors. Hence, our results aim towards a targeted product design to provide tailored silver–gold alloy NPs without the need of complex NP synthesis routes while maintaining green synthesis conditions. We further intend to establish our synthesis protocol as a model system for automated syntheses and for the detailed investigation of the particles' formation mechanism.

## 2 Materials and methods

### 2.1 Synthesis of silver–gold alloy nanoparticles

Silver nitrate in aqueous solution (1 M) was purchased from VWR Chemicals (Darmstadt, Germany). Hydrogentetrachloroaurat(iii) trihydrat was purchased from Sigma Aldrich (Taufkirchen, Germany). Sodium hydroxide solution (1 M) was purchased from Honeywell (Seelze, Germany). Dextran with a molecular weight of 40 kDa was purchased from ITW Reagents (Darmstadt, Germany). All reagents have been used without further purification. Deionized water (>17 MΩ cm) was used for all experiments in this work.

For the synthesis of alloy NPs with a molar gold content of 50%, a dextran solution was prepared by dissolving 5 g of dextran in 100 mL of water. 4.5 mL of this solution was transferred into a 10 mL snap cap vial and mixed with 0.5 mL of a 1 mM aqueous solution of AgNO_3_ and 0.5 mL of a 1 mM aqueous solution of HAuCl_4_. Different compositions were produced by changing the molar ratio of silver to gold, while keeping the total volume of 1 mL constant. After mixing, 0.5 mL of a 10 mM NaOH solution was added to the mixture to start the reduction, followed by another mixing step. The mixing was stopped and the solution was kept under the fume hood overnight. After several hours, the solution showed an orange color, which can be attributed to the formation of stabilized silver–gold alloy NPs. Pure gold NPs were synthesized according to the same protocol, while for the synthesis of silver NPs, the concentration of the NaOH solution was set to 100 mM.

### 2.2 Methods

#### 2.2.1 UV-vis spectroscopy

UV-vis spectroscopy was performed using the Varian Cary 100 spectrophotometer (Waldbronn, Germany) for a wavelength range of 200 nm to 800 nm. The spectral resolution was set to 1 nm. All samples were measured against a reference sample containing water as the solvent used in the synthesis. Single-use plastic UV cuvettes with an optical path length of 1 cm were used for all measurements. All samples were taken directly from the synthesis without any further dilution or purification.

#### 2.2.2 Analytical ultracentrifugation

Following the protocol from Cardenas Lopez *et al.*,^58^ a modified preparative ultracentrifuge, type Optima L-90 K, from Beckman Coulter (Krefeld, Germany) equipped with a multi-wavelength extinction detection system from Nanolytics Instruments (Potsdam, Germany) was used for gravitational sweep and sedimentation velocity MWL-AUC experiments. Details about the applied multi-wavelength optics and data acquisition can be found in the literature.^59,60^ The samples were taken directly from synthesis without further purification. Samples with a maximum extinction (path length 12 mm) at the LSPR wavelength < 1 were analyzed without additional dilution, while samples with an extinction value > 1 were diluted with the dextran solution used during the synthesis to a value below unity. Instability upon dilution was not observed. For gravitational sweep experiments, titanium centerpieces with an optical path length of 12 mm were used. For the sedimentation velocity experiments, 3D printed centerpieces with an optical path length of 12 mm were employed. All experiments were conducted at 20 °C. Gravitational sweep sedimentation data was acquired in the following manner. First, the rotor speed was set to 1500 rpm for an initial stage of one minute. Then, the rotor speed was slowly increased to a final speed of 35 000 rpm and held for the necessary amount of time until all NPs had sedimented. The sedimentation velocity experiments were conducted with a radial step size of 50 μm and rotor speeds of 5500 rpm (silver and gold NPs) and 18 000 rpm (silver–gold alloys).

The acquired gravitational sweep sedimentation data was analyzed using HDR-MULTIFIT (high dynamic range-multi-wavelength fitting)^61^ with 150 grid points and a confidence level of 0.95. Sedimentation velocity data of the measured silver and gold NPs was analyzed with a *c*(*s*) continuous size distribution model in the software Sedfit (version 16-1c).^62^ The data was fitted with the second derivative regularization using a confidence level of 0.95 and resolution of 150 grid points. In addition, automated multi-wavelength analyses were conducted for selected alloy NPs using a modified ls-g*(*s*) algorithm based on the work of Walter *et al.*^63^ Data was fitted with the second derivative regularization using a confidence level of 0.9 as well as 200 grid points for the sedimentation coefficient and 0.5 nm resolution for the wavelength. Multi-wavelength sedimentation coefficient distributions obtained from gravitational sweep and sedimentation velocity data were analyzed using the optical-back coupling (OBC) method according to the procedure of Cardenas *et al.*^58^

As relevant input for the AUC and OBC analyses, the dynamic viscosity and density of the solvent were measured at 20 °C with a Lovis 2000 ME viscosimeter and a DMA 5000 M density meter, respectively. Both instruments from Anton Paar (Graz, Austria). A medium viscosity of 2.276 mPa s and a density of 1.01673 g cm^−3^ were measured.

#### 2.2.3 Analytical high pressure liquid chromatography

Chromatographic experiments were performed on an Ultimate 3000 UHPLC setup (Thermo Fisher Scientific, Waltham, MA, USA) including a solvent rack (SR-3000), a quaternary pump (LPG-3400SD), an autosampler (WPS-3000SL), a column thermostat (TCC-3000RS) and a diode-array detector (DAD-3000). Analysis was performed under a constant temperature of 25 °C and a flow rate of 0.5 mL min^−1^ using a Reprosil Saphir column (300 × 8 mm) with 10 μm unmodified silica particles and a mean pore size of 100 nm. An aqueous solution of 2 mM SDS and 8 mM ammonium acetate solution was used as mobile phase. 30 μL of all samples were injected after the synthesis without any further purification. Chromatograms were measured at a wavelength of 450 nm.

#### 2.2.4 Scanning transmission electron microscopy (STEM)

STEM measurements were performed in a GeminiSEM 500 from ZEISS (Wetzlar, Germany), equipped with a STEM detector and operated at an acceleration voltage of 30 kV. Prior to the experiments, the particles were washed with and redispersed in Millipore water and subsequently dispersed on a carbon film on carrier mesh copper TEM grid, 200 mesh (Plano GmbH, Wetzlar, Germany). Particle size distributions were obtained from manual measurement of the particles' diameter from images of this instrument using the software “ImageJ”.

#### 2.2.5 High resolution scanning transmission electron microscopy (HR-STEM) and energy dispersive X-ray spectroscopy (EDX)

The HR-STEM in high-angle annular dark-field (HAADF) mode and STEM-EDX (referred to as EDX in the text) analyses were performed using a double aberration-corrected FEI Titan Themis^3^ 300 transmission electron microscope equipped with a Super-X EDX detector and operated at an acceleration voltage of 300 kV. The analyses were carried out at a convergence angle of 15.7 mrad, a dwell time of 50 μs and a screen current of around 200 pA. For all three compositions, at least six nanoparticles were analyzed in at least two independent EDX maps. The evaluations were performed using Velox version 3.0.0.815 software from Thermo Fisher Scientific, with background correction and fitting of the respective elements. To determine the molar content, the arithmetic mean of each element of the measured particles was calculated with the corresponding standard deviation. Prior to the experiments, the alloy NPs were washed with and re-dispersed in Millipore water. Next, the sample dispersion was dried on an ultrathin carbon film on a Lacey carbon support film copper TEM grid, 400 mesh (Ted Pella, Inc.) and cleaned in a Fischione Model 1020 plasma cleaner, with a gas mixture of 75% argon and 25% oxygen, for 10 seconds.

#### 2.2.6 Inductively coupled plasma-optical emission spectroscopy (ICP-OES)

Prior to all measurements *via* ICP-OES, the alloy NPs were precipitated with ethanol and washed multiple times. The precipitate was dissolved in aqua regia. All ICP-OES measurements were carried out using the Optima 8300 ICP-OES by PerkinElmer.

#### 2.2.7 Mie theory calculations

All Mie simulations have been carried out using the algorithm, which has been initially described by Wiscombe.^64^ In this work, a code by Jan Schäfer from the University of Ulm^65^ has been modified, which is based on the book of Bohren and Huffman.^66^ The code was written and compiled with MATLAB (Version 2020A). Mie calculations have been performed to validate the optical model and thereby calculate the composition of the produced alloy particles based on their optical response. The refractive index was taken from literature^67^ and interpolated on a finer grid in terms of alloy composition. Moreover, due to the reduced size of the alloy particles, corrections of the dielectric function were applied. The description of this metal cluster size effect is well-described elsewhere.^68–71^ In short, the reduced mean free path of electrons in the metal particle leads to an increase of collision time, hindering electron relaxation. To account for this effect, the damping constant takes the form *γ*_NP_ = *γ*_bulk_ + 2*Cv*_F_/*x*.^72^ Following the protocol from Cardenas Lopez *et al.*,^58^ we obtain *C*_Ag_ = 0.2959 and *C*_Au_ = 0.2035. For alloy particles, we assume a linear dependency of *C* with the molar gold content. A detailed comparison of calculated and experimentally acquired optical spectra can be found in the ESI.†

## 3 Results and discussion

### 3.1 Synthesis of pure silver and gold nanoparticles

**Fig. 1:**
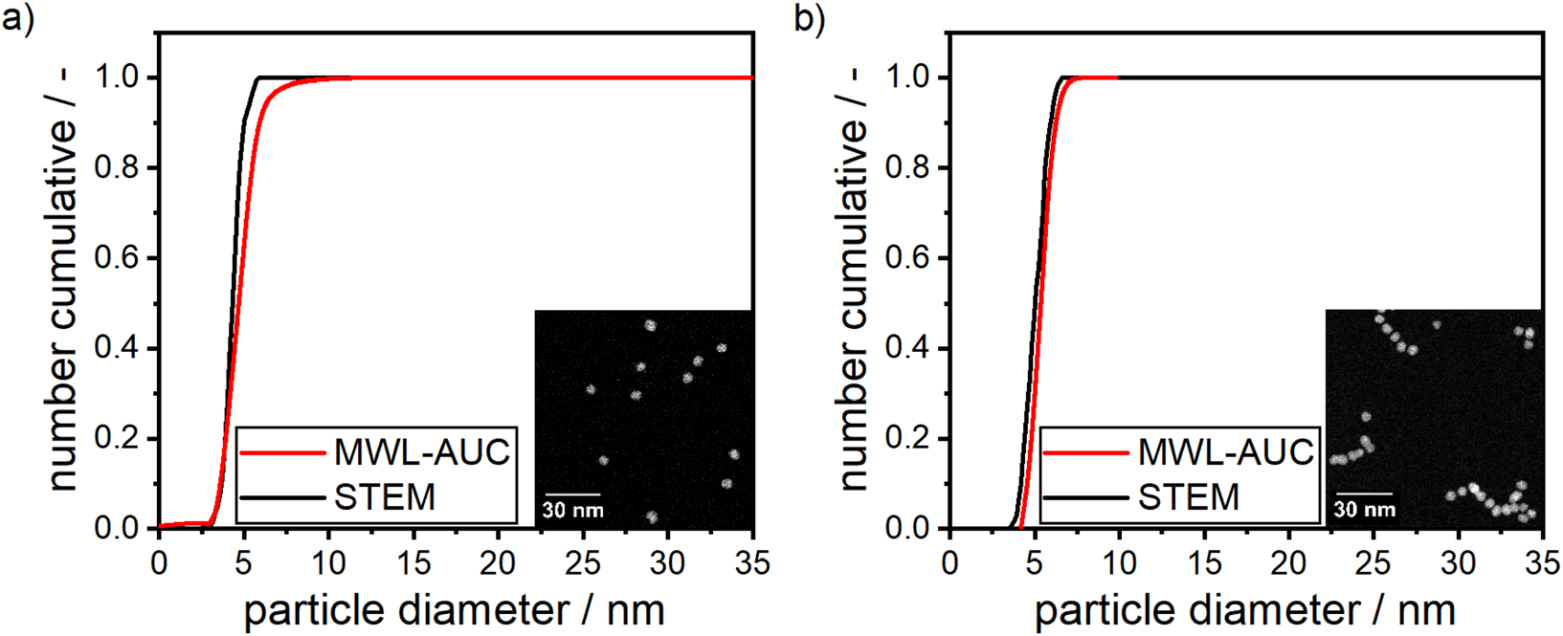
STEM images and measured PSDs of silver (a) and gold (b) NPs produced according to the experimental procedure described in the materials and methods section.

The synthesis of pure silver and gold NPs using dextran as the reducing and the stabilizing agent has been reported in literature before.^33,57,73^ As noted, the resulting particles under the described conditions show a fairly wide PSD^33,73^ or strong agglomeration within the samples,^57^ hence in our work, synthesis conditions were optimized to gain an understanding of the system and receive NPs with a mean size below 10 nm with a narrow PSD. For this purpose, preliminary experiments were performed on the synthesis of pure silver and gold NPs prior to the synthesis of silver–gold alloy NPs. The synthesis protocol was carried out according to the methodology, which has been outlined in the materials and methods section of this publication. The optimized conditions were found to be at pH 10 and 12 for gold and silver NPs, respectively, room temperature and the concentrations described in the materials and methods section of this work. The inset of Fig. 1 depicts STEM images of synthesized silver NPs (Fig. 1a) and gold NPs (Fig. 1b) under optimized synthesis conditions, serving as a proof-of-concept for our synthesis of spherical NPs. Moreover, Fig. 1 shows the PSD as measured by MWL-AUC experiments and validated by STEM image analysis (>160 particles counted). Both PSDs agree very well.

Furthermore, it can be seen from both panels in Fig. 1 that the NPs show a narrow PSD with a mean particle size of (4.47 ± 0.56) nm for Ag and (5.1 ± 0.67) nm for Au, respectively, as retrieved from the MWL-AUC distributions. The standard deviations are 12.7% and 13.1% for silver and gold, respectively, which is in line with standard syntheses for gold and silver NPs found in literature. Puntes *et al.* reported values of 9.7% (ref. 74) and 11.9% (ref. 75) for silver and gold respectively, while Turkevich reported values between 8.1% and 12.5% (ref. 76) for his standard gold NP synthesis. A stabilizing dextran shell with a thickness of 2.3 nm was retrieved from sedimentation velocity experiments and assumed constant for all compositions of particles between pure silver and gold. Details on the method to determine the shell thickness can be found in the ESI.† Notably, the pH during the synthesis of the silver NPs was slightly higher. This is firstly due to the formation of Ag_2_O from AgNO_3_ under alkaline conditions,^33,51,77^ which shows a faster reduction kinetic to Ag^0^ in the subsequent step than the reduction of silver ions.^51,77^ It was shown by Nishimura *et al.* that an increasing ratio of NaOH to AgNO_3_ increases the reaction kinetics for the formation of Ag^0^.^77^ Secondly, an increase in the pH increases the potency of dextran due to the formation of alkoxide groups. Alkoxide groups are generated through the deprotonation of hydroxyl or aldehyde groups by the negatively charged hydroxide ions in solutions at elevated pH levels. The negatively charged alkoxide groups subsequently reduce the metal ions and are therefore responsible for the reduction of metal ions to metal atoms.^33,78^ Both effects thereby lead to a higher supersaturation of silver atoms, which in turn leads to a faster nucleation step, resulting in a higher concentration of smaller silver particles and a narrower distribution in sizes.^79^

### 3.2 Synthesis and characterization of silver–gold alloy NPs

#### 3.2.1 Synthesis and characterization of size and composition

**Fig. 2:**
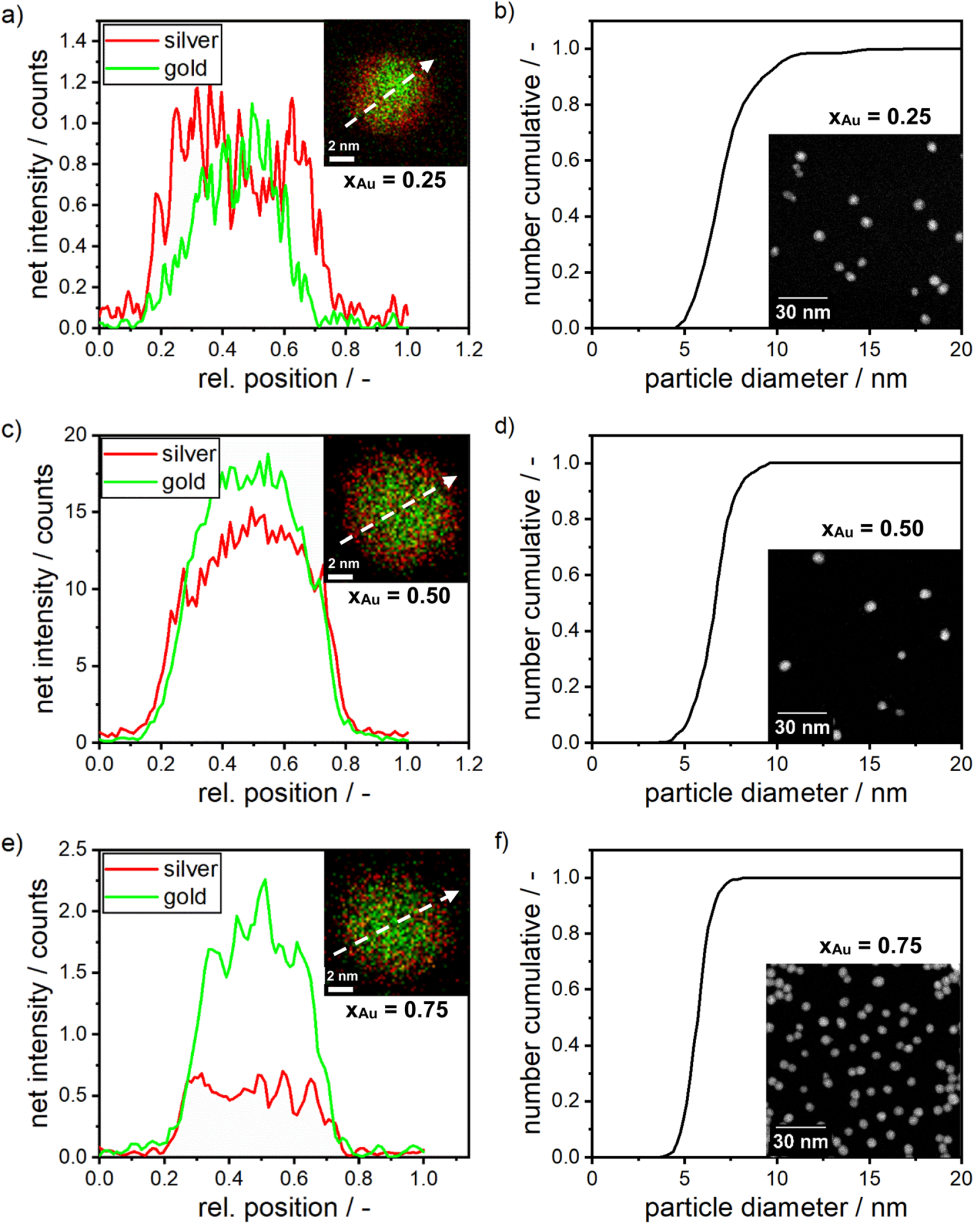
(a) EDX map of silver–gold alloy NPs with a molar gold content of 25% (inset) and their respective elementary concentration over the spatial coordinate. (b) PSD for a molar gold content of 25% as retrieved from STEM measurements and exemplary STEM images (inset). (c) EDX map of silver–gold alloy NPs with a molar gold content of 50% (inset) and their respective elementary concentration over the spatial coordinate. (d) PSD as retrieved from STEM measurements and exemplary STEM images (inset). (e) EDX measurement for alloy NPs with a molar gold content of 75% (inset) and their respective elementary concentration over the spatial coordinate. (f) PSD for a molar gold content of 75% as retrieved from STEM measurements and exemplary STEM images (inset). The EDX maps show the net intensity of gold in green and silver in red with the white arrow indicating the line scan, measuring the local element distribution shown in graph a, c and e. Additional HR-STEM images of the samples can be found in Fig. S7 in the ESI.†

Following the synthesis of silver and gold NPs using dextran as the reducing and capping agent, silver–gold alloy NPs with a broad range of molar compositions were synthesized at room temperature in order to demonstrate the wide applicability of our method. As a first validation step for our successful synthesis of homogeneously alloyed NPs, the alloy NPs were analyzed with respect to chemical composition and PSD using STEM and STEM-EDX measurements. The STEM analysis proves a narrow PSD with median particle sizes between 5 and 8 nm (see Fig. 2b, d and f). Moreover, STEM-based EDX analysis was performed over the spatial coordinate of a single NP, which is demonstrated in Fig. 2a, c and e.^80^

**Fig. 3:**
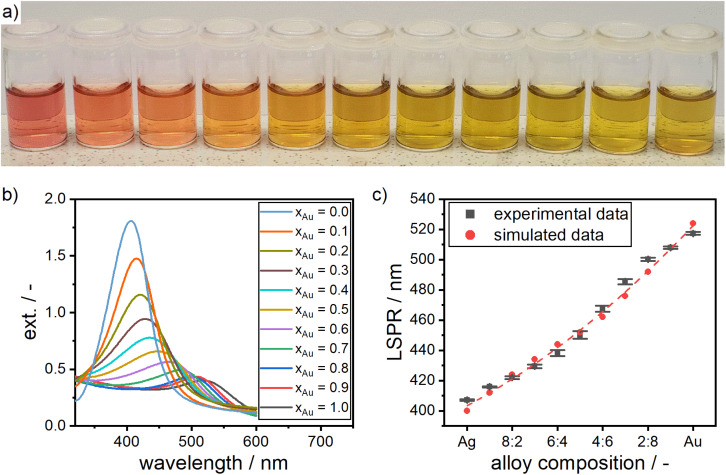
(a) Silver–gold alloy NP dispersions from pure gold (left) to pure silver (right) in 10% composition intervals. (b) UV-vis extinction spectra of silver–gold alloy NPs and (c) their LSPR compared to the simulated values using Mie's theory calculations.

The analysis of the EDX measurements shows the chemical composition for alloy NPs with an expected molar gold content of 25%, 50% and 75%. Mean molar gold contents of (24.5 ± 7.5)%, (47 ± 2)% and (79 ± 7.0)% are retrieved respectively, which is in good agreement with the targeted values and proofs that all compositions can be synthesized within good accuracy with our protocol. This result validates that the composition of the alloy NPs can be controlled by the initial molar concentration of the precursors during a room temperature synthesis using dextran as a reducing and capping agent. Furthermore, the line profiles through the particles in the EDX analyses proof that the molar distribution within an alloy NP is, in most cases, homogenous. However, as typical for co-reduced silver–gold bimetallic NPs, some of the produced NPs, especially NPs with a high molar silver content (Fig. 2a, silver content = 75%), show an enrichment of gold in the center of the particles and an enrichment of silver atoms towards the outer spatial coordinate of the NP.^39,44,81,82^ This can be explained by the higher bond strength between gold atoms, compared to the gold–silver bond, which is higher than the bond between silver atoms.^56^ Also the redox potential of gold ions is higher compared to silver ions.^83–85^ Finally, the surface energy of silver is lower than that of gold.^56^ While a slight gradient of the composition seems to be present within some of the particles, no core–shell structure was found. The analysis of the optical properties of the alloy NPs (see Fig. 3) further proofs that point.

#### Characterization of optical properties

With the control over the chemical composition well ensured, the analysis of the resulting optical properties of the alloy NPs is essential for targeted product design. In this context, Link *et al.* showed that the localized surface plasmon resonance (LSPR) of silver–gold alloy NP suspensions shifts linearly between the LSPR of silver particle suspensions at a wavelength of 400 nm and the LSPR of gold NP suspensions at a wavelength of 520 nm.^42^ Core–shell structures of the same elements, however, exhibit multiple peaks in the UV-vis spectrum^86,87^ or a completely different peak shape.^58^ Thus, the analysis of the optical properties is the ideal tool for further validation of our synthesis route.

In order to proof the accuracy with which the composition of the produced particles can be adjusted using our new synthesis route, we synthesized silver–gold alloy NPs for a broad range of molar gold contents in intervals of 10 mol%. The alloy NP suspensions show a color spectrum in between bright red, which is typical for gold NPs, and bright yellow, which is typical for silver NPs, according to their molar composition (see Fig. 3a). Remarkably, the corresponding UV-vis spectra (Fig. 3b) show only one peak, which is unequivocally distinguishable for each composition, clearly demonstrating the precise adjustability of the particles' compositions.

To further solidify the optical model and create a basis on which the composition of the synthesized alloy NP can be assessed, ICP-OES measurements were performed for alloy NPs with a molar gold content of 26%, 58% and 82% according to the optical model. The ICP-OES measurements confirmed the molar gold content of the alloy NPs, which was determined from the optical model after Rioux^67^ (Table 1). This proofs that the used optical model is in fact accurately predicting the molar composition of the produced silver–gold alloy NPs. Slight deviations in the retrieved compositions can be explained by minor variations in the preparation of the ICP-OES samples as well as the measurement itself.

**Table 1:** Molar gold and silver contents retrieved from ICP-OES measurement results for three selected alloy compositions and compared with the optical model

LSPR position/nm	Gold concentration from ICP-OES measurements/mg L^−1^	Silver concentration from ICP-OES measurements/mg L^−1^	Molar silver content retrieved from ICP-OES measurements/—	Molar silver content retrieved from optical model/—
430	4.80 ± 0.30	6.04 ± 0.18	0.70 ± 0.009	0.74
464	8.24 ± 0.37	2.98 ± 0.13	0.40 ± 0.001	0.42
500	15.02 ± 0.32	2.04 ± 0.10	0.20 ± 0.005	0.18

As expected and described above, the spectrum in the color of the solutions results from a shift in the plasmonic peak between the LSPR of silver and gold NPs. Simulations using Mie's theory further confirmed the shift in the LSPR. Fig. 3c shows the shift in the LSPR according to theoretically modeled values from Mie calculations, compared to the measured values. The UV-vis spectra are in good agreement with the simulated LSPR values. This indicates that the formation of alloy NPs with a highly controllable composition is possible by our synthesis protocol.

The theoretical LSPR positions over the respective alloy composition can be fitted *via* a quadratic function (see eqn (1) and the dashed line in Fig. 3c). Using the function, the LSPR position of alloys with any given molar gold content *x*_Au_ can be calculated.

1 LSPR/nm = 403.0 + 87.10*x*_Au_ + 29.72*x*_Au_^2^

The equation is a purely empirical fit, which is only valid for particles of the same size as the particles within this study (5–8 nm), since the plasmon bands shift to higher wavelengths for larger particles.

#### 3.2.2 Investigation of distributions in size and molar composition

**Fig. 4:**
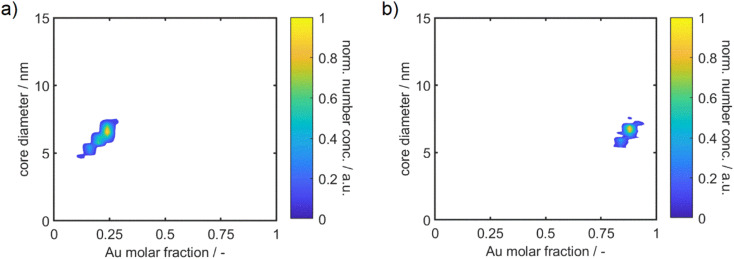
Two-dimensional property distributions of the synthesized alloy NP dispersions with a molar gold content of 25% (a) and 90% (b). Additional distributions can be found in the ESI.† The particle size is displayed on the *y* – axis and the molar gold content on the *x* – axis.

As microscopy image analysis and auxiliary EDX measurements only allow for the analysis of a limited number of NPs, we analyzed the NP suspensions *via* MWL-AUC experiments. Recently, Cardenas Lopez *et al.*^58^ demonstrated an experimental protocol, which is based on coupling hydrodynamic and optical properties of alloy NPs, in order to measure 2D distributions with respect to NP size and composition from a single MWL-AUC measurement. In short, the optical properties of the particles with a certain sedimentation coefficient interval are retrieved. The measured spectra for each interval are subsequently fitted by a multitude of theoretical single-species spectra calculated by Mie's theory. This OBC technique was applied to a series of silver–gold alloy NPs. The resulting two-dimensional distributions of suspensions with a molar gold content of 25% and 90% are provided in Fig. 4. The two-dimensional distributions for a broader range of compositions are provided in the ESI in Fig. S3.† Furthermore, the results from MWL-AUC data allow independent validation of the PSDs retrieved by STEM. Overall, distributions by STEM and AUC show good agreement as exemplarily shown in the ESI, Fig. S2b.† Samples with a higher silver content than 60% were measured 72 h after the synthesis, since a ripening effect with parallel mixing of silver and gold atoms has been observed (see Fig. S4†). A detailed investigation of the formation mechanism is currently conducted within our group. Remarkably, the NP size and composition is narrowly distributed and the NP size stays constant over the different compositions. Moreover, aggregation of alloy NP suspensions and any segregation during the synthesis, *i.e.* the formation of multiple species, can be excluded based on the results from MWL-AUC. This underlines the use of silver–gold alloy NPs for optimization of macroscopic optical properties with well-ensured colloidal stability as well as the value of the performed MWL-AUC experiments.

**Fig. 5:**
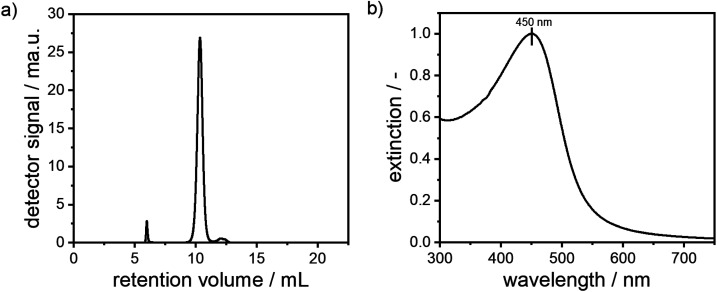
Chromatogram and corresponding UV-vis extinction signal of a suspension of silver–gold alloy NPs with a molar gold content of 50%.

In order to confirm the presence of a narrow monomodal PSD as retrieved from MWL-AUC and to investigate colloidal stability with respect to particle–particle interactions within the dispersions, size-exclusion chromatography (SEC) was performed. Here, the retention volume is coupled with the hydrodynamic diameter of the alloy NPs. While smaller NPs enter the pores of the stationary phase materials more deeply and therefore have a longer residence time within the column, larger NPs are restricted to larger pores only, thus exit the column faster. The zeta potential of the synthesized particles was determined to −29 mV and the zeta potential of the stationary phase of the column was −40.7 mV. Electrostatic binding of the particles to the column can therefore be ruled out. In Fig. 5, the chromatogram of alloy NPs with a molar gold content of 50% is depicted. A narrow monomodal retention volume distribution is detected. The corresponding extinction spectra, which are directly measured during the SEC experiment, show a plasmonic peak, which corresponds to a molar gold content of 50%. A second small peak at a retention volume of around 6 mL is visible. Based on its extinction spectrum (see Fig. S5†) and its low retention volume, this can be attributed to minor agglomeration during the synthesis. Notably, due to the clear distinction of the agglomerate peak from the main signal, it can easily be separated within the SEC experiment. A third shoulder at a retention volume of 12.5 mL was identified as free unbound dextran based on its extinction spectrum (see Fig. S5†).

**Fig. 6:**
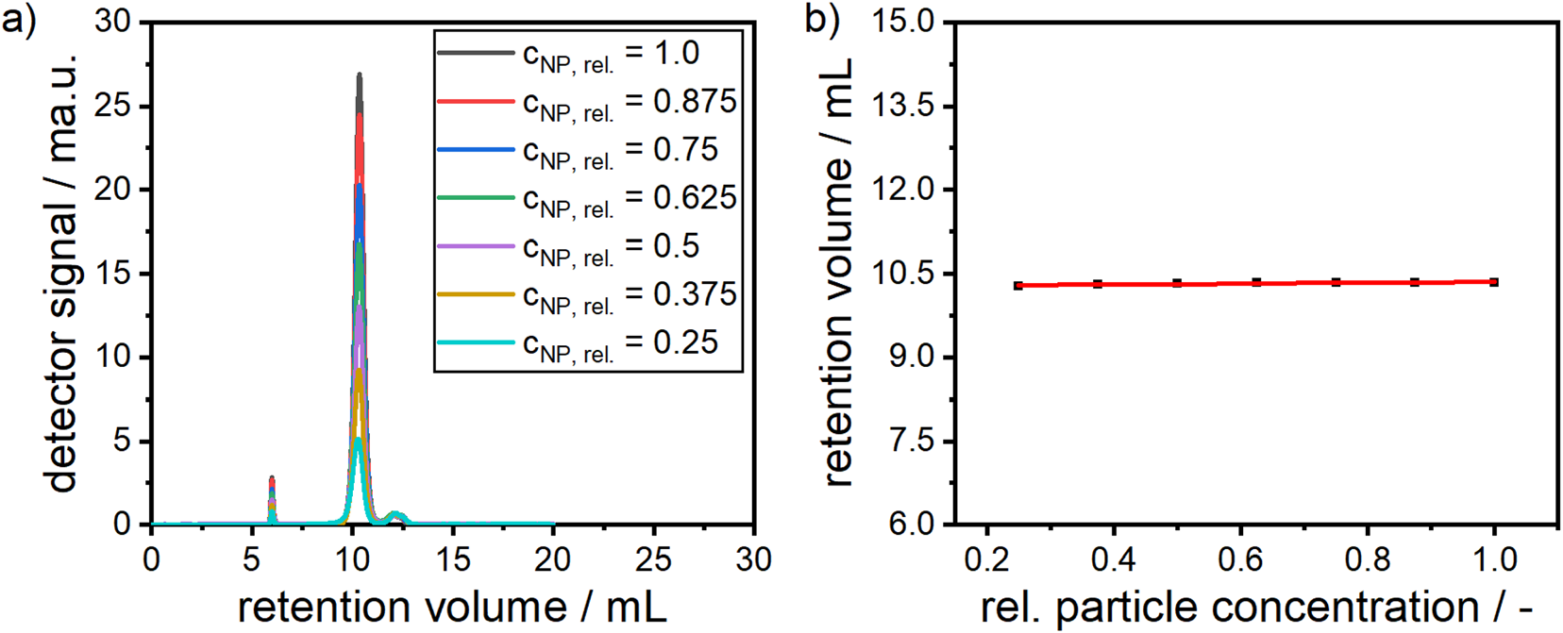
(a) Detector signals for multiple dilutions and (b) retention volume over the relative particle concentration as measured from SEC.

The stability of alloy NP dispersions is governed by particle–particle and particle–solvent interactions, which is related to concentration non-ideality^88–91^ As the alloy NPs are fully covered with a stabilizing shell of dextran, the attractive van der Waals core–core interactions are suppressed, *i.e.* the interactions are mostly defined by the interactions of the dextran shells. In order to provide insight into colloidal stability with respect to concentration non-ideality, alloy NPs with different particle concentrations were measured by SEC and the elution times were retrieved, as can be seen in both panels of Fig. 6. No significant shift in the retention volume is noticeable, which points to the excellent stability of the particles, as no agglomerates are formed and no change in the interaction with the stationary phase of the HPLC column is observed. Moreover, the peak at around 12.5 mL remains unchanged and further confirms the presence of free dextran.

#### 3.2.3 Investigation of formation kinetics

**Fig. 7:**
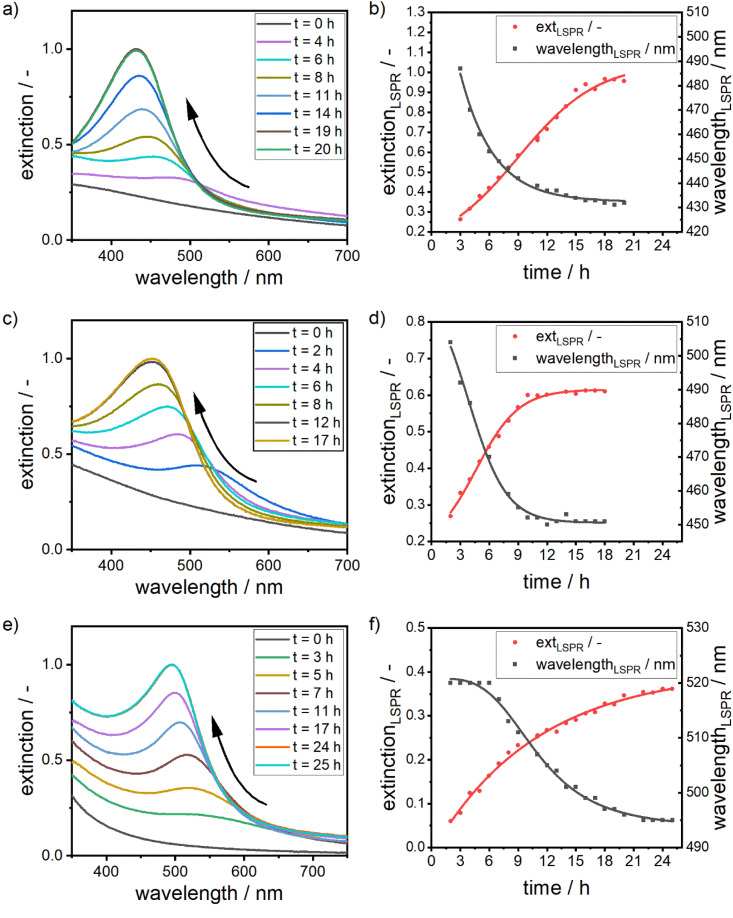
(a) Time-resolved extinction spectra of a silver–gold alloy NPs with a targeted molar gold content of 25%. (b) Corresponding shift in the LSPR position as well as the increase in the extinction at the position of the LSPR peak. (c) Time-resolved extinction spectra of a silver–gold alloy NPs with a targeted molar gold content of 50%. (d) Corresponding shift in the LSPR position as well as the increase in the extinction at the position of the LSPR peak. (e) Time-resolved extinction spectra of a silver–gold alloy NPs with a targeted molar gold content of 75%. (f) Corresponding shift in the LSPR position as well as the increase in the extinction at the position of the LSPR peak. Solid lines in (b, d and f) provide a guide-to-the-eye.

In addition to the one-step characterization *via* MWL-AUC, we further investigated the formation mechanism and kinetics of alloy NPs using UV-vis spectroscopy and the aforementioned optical model. The extinction spectra of an alloy NP dispersion with a targeted molar gold content of 25%, 50% and 75% were measured during synthesis as a function of time. As the extinction at the LSPR wavelength is connected to the NPs' size and chemical composition, it is possible to follow the formation of the NPs over time with UV-vis spectroscopy. Our results are summarized in Fig. 7.

The reaction is rather slow and appears to be completed after 17 to 24 hours (see Fig. 7b, d and f). The formation is thus reaction- and not mixing-controlled, as convection and diffusion happens on much shorter time scales. The change in the extinction values at the respective LSPR position over time can be interpreted as an effective reaction rate *r*_eff_ (see eqn (2)). For that we fit a linear equation to the region in the extinction–time diagrams (Fig. 7b, d and f), in which the extinction increases linearly with time. From the slope of this function, we can then determine the effective reaction rate (see Fig. S9 of the ESI†).2
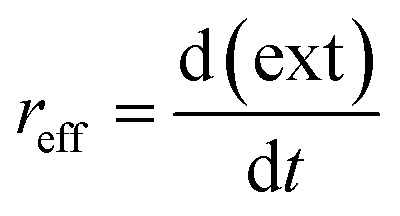


The formation of the silver–gold alloy NPs starts with the formation of a gold-rich nucleus, which grows to an alloy NP. This is associated with a shift in the chemical composition from pure gold to the desired molar gold content, which is in line with the EDX maps. Due to the higher redox potential, the gold ions are reduced faster, leading to a higher concentration of gold atoms in the solution. Together with the higher bond strength between the gold atoms, NPs, which are formed at the beginning of the reaction, show a high molar gold content.

The gold content of early particles further depends on the concentration of the gold precursor in the precursor mixture. As the early particles are formed *via* a crystallization reaction, the concentration of silver and gold atoms in solution prior to the nucleation step determines the composition of the formed NPs. This is displayed in the position of the LSPR peak at early reaction times. After three hours of reaction time, the LSPR position shifts to lower wavelengths with an increase in silver content (see Table 2). For suspensions containing NPs with a high molar gold content of 75%, the LSPR position remains at 520 nm for several hours, which is exactly the LSPR position of pure gold particles, as little silver is present in the beginning of the nucleation.

**Table 2:** Effective reaction rates *r*_eff_, LSPR positions *λ*_LSPR_ after 3 hours of reaction time, solution pH and percentage of dissolved silver in solution for suspensions containing silver–gold alloy NPs with molar gold contents of 25%, 50% and 75%

	*x*_Au_ = 0.75	*x*_Au_ = 0.50	*x*_Au_ = 0.25
*r*_eff_/h^−1^	0.025	0.04	0.05
*λ*_LSPR_ @ 3 h/nm	520	492	487
pH/—	10.91	11.08	11.17

Over time, the composition shifts and eventually reaches the desired composition. This trend is clearly visible for all three compositions, which hints at the mechanism of alloy formation.

Additionally, it can be observed that the effective reaction rates increase with an increase in silver concentration. This can be explained by a change in the pH values of the solutions. As the gold precursor used for this study is highly acidic and the concentration of NaOH is kept constant for all experiments, the pH in the reaction mixtures decreases with an increase in gold precursor concentration. As described above, the pH of the solution has a strong influence on the reduction behavior of dextran and the metal precursors:

• The reduction potential of dextran increases with increasing pH, which leads to a higher reduction rate of gold and silver.^78^

• The reduction rates of silver and gold react in an inverse fashion to an increase in the pH level. Silver shows a higher reduction rate,^77^ while gold shows a lower rate.^92^

This in sum leads to a higher effective reaction rate for silver rich alloy NPs, where the gold precursor concentration is lower and further explains the differences in the LSPR position at early time points. As the pH is higher for low gold precursor concentrations, the gold reduction rate is reduced, while the silver reduction rate is increased, which leads to a higher silver content in the early particles and thus to a lower LSPR position, further showing the extreme dependency of the system on the pH of the dispersion. Since the reaction rate captured by the change in the extinction of the system at the respective LSPR position is an effective rate, the inverse reaction of the different reaction partners to the pH value also explains the non-linear relationship between pH and effective reaction rate.

Overall, the changes in the optical spectra allow for the quantitative analysis of the formation dynamics of the particles with further *in* and *ex situ* measurement techniques and provides in-depth understanding of their formation behavior. A corresponding study is currently ongoing. Notably, our results are directly in accordance with findings reported in the literature over a variety of different reaction routes.^39,44,49^

#### 3.2.4 Scale-up

In order to move from a fundamental understanding of the formation of NPs to large scale production, the potential for scale-up needs to be explored. Scale-up can be performed either by an increase of reactor volume or by an increase of particle concentration within the dispersion. We investigated the potential of scale-up in both ways using UV-vis spectroscopy and SEC.

**Fig. 8:**
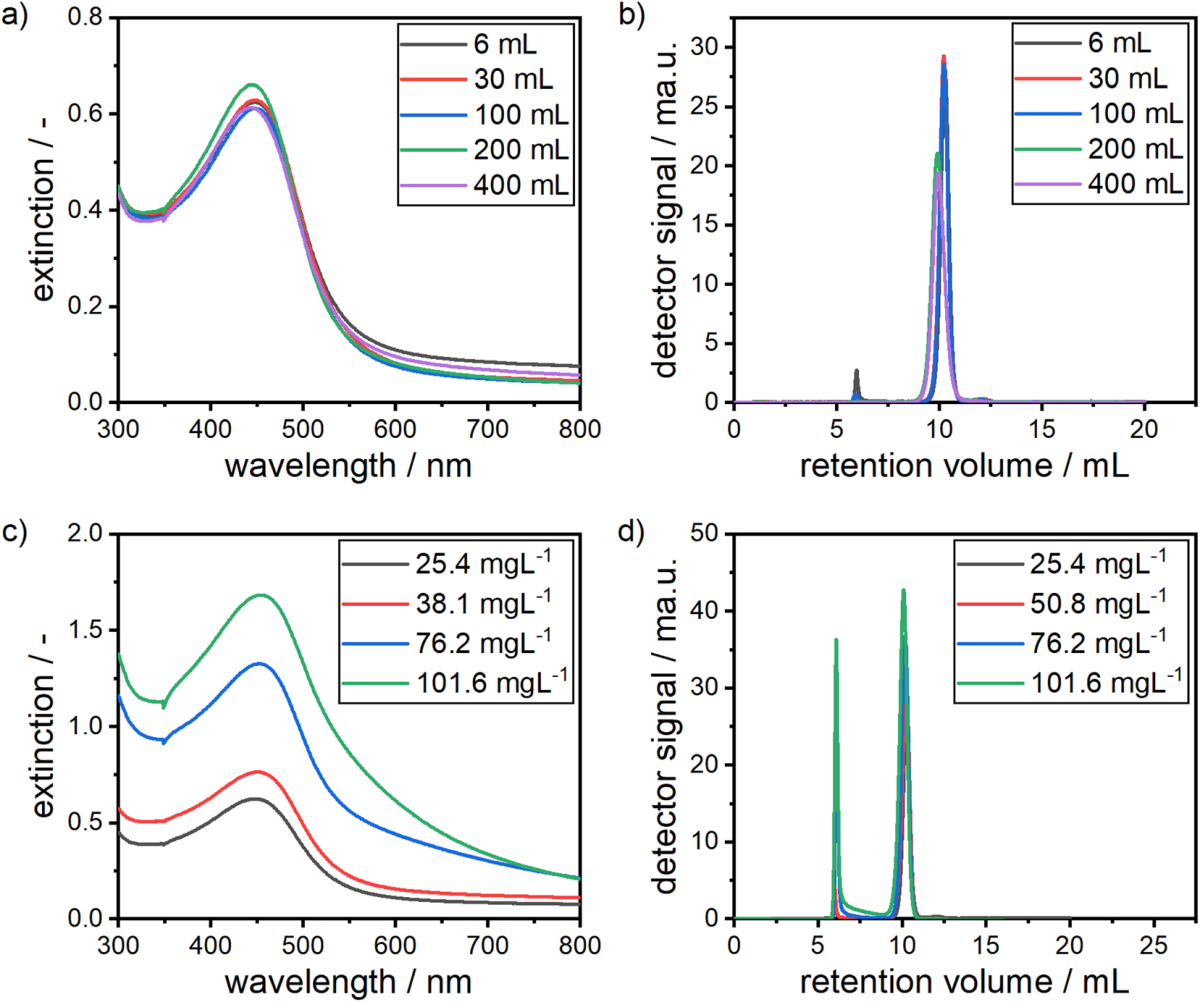
UV-vis spectra of alloy NP dispersions produced at varying reaction volumes between 6 mL and 400 mL (a) and their corresponding chromatograms (b). (c) UV-vis spectra of alloy NP dispersions with varying NP concentrations between 25.4 mg L^−1^ and 101.6 mg L^−1^ and their corresponding chromatograms (d).

As the herein described formation process of alloy NPs is reaction controlled, mass transfer issues during mixing are avoided when moving from small to larger reactors. Fig. 8a and b show the results for an increase in reaction volume by a factor of more than 60 from an initial volume of 6 mL to a maximum volume of 400 mL.

Remarkably, the measured dispersions show almost identical optical spectra and retention volume distributions. Slight differences, especially in the retention volumes, which would indicate a slight shift to larger particles, can be explained by small differences in the precursor batches. The first batch was used for volumes of 6 mL to 100 mL and another batch for volumes of 200 mL and 400 mL. This clearly shows that no mixing effects are present and that a scale-up to larger reactors independent of their size is easily possible with the presented synthesis approach, as long as the mixing of the precursors is way faster than the formation kinetics of the particles, which is clearly the case under the conditions studied.

Fig. 8c and d show the system's response to an increase in the precursor concentrations by a factor of 4 from 25.4 mg L^−1^ to 101.6 mg L^−1^. It should be noted that the concentration of the added sodium hydroxide solution was stepwise adjusted from the initial 10 mM to 30 mM for the highest precursor concentration in order to account for the low pH of the gold precursor. It can be clearly seen that the position of the LSPR peak in the UV-vis spectrum stays at a constant position, showing that the chemical composition of the particles remains constant, given that the size of the particles also remains in the same range, which can be seen in the chromatograms in Fig. 8d. At larger wavelengths of the extinction spectra, a shift to higher extinction values can be observed, which indicates the formation of larger amounts of agglomerates. This can be confirmed from the chromatograms, which show an increase in the agglomerate peak at retention volumes of around 6 mL with an increase of precursor concentration. This means that the quality of the produced particle dispersions suffers at higher concentrations and the effort in purification increases.

In summary, the combined increase in reactor volume and NP concentration would lead to a scale-up by factor of more than 250. Utilizing techniques like SEC, agglomerates formed at higher particle concentrations can be easily separated to keep the product quality constant.

Established syntheses for monometallic NPs reach particle concentrations in the range of 30 mg L^−1^. Some examples are the works of Puntes *et al.*, who received concentrations of 26.7 mg L^−1^ and 32.6 mg L^−1^ for citrate based syntheses of silver^74^ and gold^75^ particles, respectively. The original paper by Turkevich reports particle concentrations of 29 mg L^−1^.^76^ Our synthesis shows values between 25.4 mg L^−1^ and 101.6 mg L^−1^. It must be noted, however, that these concentrations are given for monometallic NPs. For the production of silver–gold alloy NPs, the solubility of AgCl has to be taken into account, which is why the concentrations are usually chosen lower. Link *et al.* performed the Turkevich synthesis for alloy NPs and received mass concentrations of 22.43 mg L^−1^,^42^ which is already lower than in our synthesis. Here it should be noted that the synthesis is performed at 100 °C, which significantly improves the solubility of the AgCl. Mallin *et al.* present a typical room temperature synthesis using NaBH_4_ as the reducing agent. They receive particle concentrations of only 0.8 mg L^−1^ (ref. 45) due to the extremely low solubility of AgCl at room temperature. The thereby realized particle concentrations are lower than the ones obtained in this manuscript by a factor of more than 30, which clearly shows the unprecedented advantage of our synthesis route.

## 4 Conclusion

In our manuscript, we present a green room temperature synthesis of silver and gold NPs and their homogenous alloys using dextran as the reducing and capping agent. Our synthesis produces NPs with a mean size of 5 nm to 8 nm with narrow size distribution and precisely adjustable molar gold content. The macroscopic optical properties of the alloy NPs are directly controlled by the alloy composition. While the mean particle size remained constant over a broad range of alloy compositions, the molar composition was validated using STEM-EDX as well as auxiliary ICP-OES measurements. With the composition of the alloy NPs well ensured, the predictions from optical modelling by Mie's theory matched our experimental results. Moreover, the distributions in size and chemical composition were analyzed *via* MWL-AUC experiments demonstrating that the distributions in the molar composition of our alloy NPs are quite narrow. The particle evolution kinetics was evaluated indicating that the synthesis is reaction-controlled allowing for relatively easy scale-up. Our study showcases an environmentally friendly and scalable synthesis for metallic alloy NPs, thus avoiding the need for harsh process conditions while maintaining the high quality of the produced particles.

## Conflicts of interest

There are no conflicts to declare.

## Supplementary Material

NA-005-D2NA00793B-s001
