# Supplementary material for: Green room temperature synthesis of silver–gold alloy nanoparticles
Source: Nanoscale Adv. 2023 Feb 13;5(5):1450–64. doi: 10.1039/d2na00793b (PMC9972530; doi:10.1039/d2na00793b)
Supplement: NA-005-D2NA00793B-s001 [file NA-005-D2NA00793B-s001.pdf]

Electronic Supplementary Material (ESI) for Nanoscale Advances.
This journal is © The Royal Society of Chemistry 2023

Electronic Supporting Information
Green room temperature synthesis of silver-gold alloy nanoparticles

N. E. Traoré[a,b], M. J. Uttinger[a,b], P. Cardenas Lopez[a,b], D. Drobek[c], L. Gromotka[a,b], J. Schmidt[a,b], J. Walter[a,b], B. Apeleo Zubiri[c], E. Spiecker[c], W. Peukert[a,b]

a) Institute of Particle Technology, Friedrich-Alexander-Universität Erlangen-Nürnberg, Cauerstraße 4, 91058 Erlangen, Germany

b) Interdisciplinary Center for Functional Particle Systems, Friedrich-Alexander-Universität Erlangen-Nürnberg, Haberstraße 9a, 91058 Erlangen, Germany

c) Institute of Micro- and Nanostructure Research (IMN) & Center for Nanoanalysis and Electron Microscopy (CENEM), Interdisciplinary Center for Nanostructured Films (IZNF), Friedrich-Alexander-Universität Erlangen-Nürnberg, Cauerstraße 3, 91058 Erlangen, Germany

*Corresponding author: W. Peukert: wolfgang.peukert@fau.de

Optical Modelling

In order to firstly have an indication of the successful synthesis of a desired alloy composition and secondly accurately determine particle size and composition using hydrodynamic and spectral data retrieved from analytical ultracentrifugation (AUC) experiments, optical modelling of alloy particles' extinction spectra must be as accurate as possible. Mie theory can be applied to determine these spectra. Figure S1 shows a comparison of the measured and the calculated extinction spectra, after fitting for the empiric dampening coefficient, for three exemplary alloy compositions.

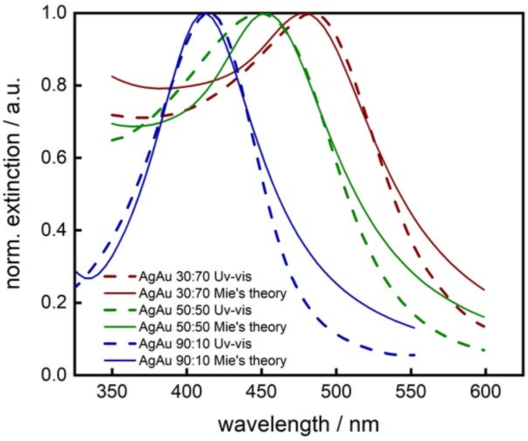

Figure S1: Normalized simulated extinction spectra of selected silver-gold alloy NPs in comparison to their experimental spectra.

Back in 1908, Gustav Mie[1] considered the interaction of an electromagnetic wave with a solid, spherical, isotropic and smooth particle. In his theory, the complex dielectric functions of the surrounding medium and the homogeneous particle are assumed to be continuous and isotropic. The incident wave, the wave within the body and the

scattered wave follow the same fundamental Maxwell equations.[2] The following section presents the basic relations of Mie's theory, which are relevant to this work.

The extinction cross-section C_{ext} of a smooth, isotropic and spherical particle is defined as the sum of the absorbance cross-section C_{abs} and the scattering cross-section C_{sca}:

$$C_{ext} = C_{abs} + C_{sca} \tag{1}$$

The extinction efficiency Q_{ext} is the ratio of the extinction cross-section and the projected area A_P of the respective particle:

$$Q_{ext} = \frac{C_{ext}}{A_P} \tag{2}$$

The extinction cross-section can further be derived from the Mie-coefficients, which depend upon the NPs' size, wavelength and the complex refractive indices, which are material- and wavelength-dependent.[3] In the case of monodisperse NPs, the turbidity τ_λ is linked to the extinction cross-section of a single particle via the number concentration of the NPs c_{NP}:

$$\tau_\lambda = c_{NP} \cdot C_{ext} \tag{3}$$

Combining Equations 2 and 3 gives:

$$\tau_\lambda = \frac{3 c \cdot Q_{ext}}{2\ \ d\rho_P} \tag{4}$$

By rearranging Equation 4, an expression for the specific turbidity τ_λ/c, i.e. the wavelength-dependent extinction coefficient ε_λ, is formulated:

$$\frac{\tau_\lambda}{c} = \varepsilon_\lambda = \frac{3\ Q_{ext}}{2d \cdot \rho_P} \tag{5}$$

Wiscombe developed an algorithm for the calculation of the extinction coefficient for given material and optical parameters.[4] The size parameter χ, which relates the size of the NPs x to the wavelength of the light λ, serves as an input to the algorithm:

$$\chi = \frac{\pi \cdot x}{\lambda} \tag{6}$$

Additionally, the refractive indices of both, the particles and the surrounding medium, need to be known.

Analytical Ultracentrifugation

The sedimentation coefficient s of a nanoparticle (NP) is defined as the sedimentation velocity u normalized to the centrifugal field $\omega^2 r$. It is further defined by the mass of a particle m_P, its partial specific volume $\bar{v}$ and translational friction coefficient f_t in case of known solvent density ρ_S:[3,5]

$$s = \frac{u}{\omega^2 r} = \frac{m_P(1 - \bar{v}\rho_S)}{f_t} \tag{7}$$

Furthermore, the diffusion coefficient D is defined by the Stokes-Einstein Equation.[6] It relates the thermal energy kT to the translational friction coefficient:[6]

$$D = \frac{kT}{f_t} = \frac{kT}{3\pi\eta x_H} \tag{8}$$

Here, the solvent viscosity is denoted as η and the hydrodynamic diameter of the NPs as x_H. During the course of AUC experiments, both sedimentation and diffusion lead to a change of the macroscopic NP concentration c over time t. This is macroscopically described by Lamm's equation, which is based on a mass-conservative approach in a sector-shaped centrifugal cell:[5]

$$\frac{\partial c}{\partial t} = D \cdot \left(\frac{\partial^2 c}{\partial r^2} + \frac{1 \partial c}{r \partial r}\right) - \omega^2 s\left(r\frac{\partial c}{\partial r} + 2c\right) \tag{9}$$

The radial coordinate within the centrifugal cell is denoted as r. Equation 9 is solved numerically in order to retrieve the diffusion-corrected sedimentation coefficient distribution.[7] The Lamm equation can further be simplified by neglecting diffusional broadening to retrieve the apparent sedimentation coefficient distribution.[8]

Core-shell analysis and shell thickness determination from sedimentation velocity AUC experiments

When NPs are dispersed in a liquid, a solvation layer is formed. Moreover, NPs are often associated with a stabilizing agent in order to ensure stability of the dispersion. Thus, the hydrodynamic diameter is calculated from the diameter of the core x_{core} and the hydrodynamic shell thickness h_{shell} according to:

$$x_H = x_{core} + 2h_{shell} \tag{10}$$

In order to account for the core-shell NPs during AUC experiments, the concept of an effective particle is applied.[9] The effective density $\rho_{P,eff}$ is directly related to the sedimentation and diffusion coefficient according to:[9]

$$\rho_{P,eff} = \rho_S + \frac{162\pi^2\eta^3 s D^2}{k_B^2 T^2} \tag{11}$$

In case of known effective density and hydrodynamic diameter, the shell thickness can be calculated according to:[9]

$$h_{shell} = \frac{x_H}{2}\left(1 - \sqrt[3]{\frac{\rho_{P,eff} - \rho_S}{\rho_P - \rho_S}}\right) \qquad 12$$

Notably, for polydisperse NPs, the effective density is a function of the particle size as the shell thickness is in good approximation constant.

In order to assess the dextran shell thickness, we conducted sedimentation velocity AUC experiments followed by a combined sedimentation-diffusion analysis using the program SEDFIT (version 16.1c). Here, we retrieved the fitted partial specific volumes of three replicates of pure gold samples. Figure S2a depicts the velocity data and residuals evaluated at 350 nm of an exemplary sample. The analysis led to a retrieved shell thickness of h_{shell} = 2.32 nm, when considering a shell composed of dextran and water in a volume ratio of 1:1. This shell thickness also led to an excellent agreement of the AUC retrieved distribution with the particle core diameter distribution measured by scanning transmission electron microscopy (STEM) as shown in Figure S2b. All further calculations are performed under the assumption that h_{shell} remains constant for all compositions.

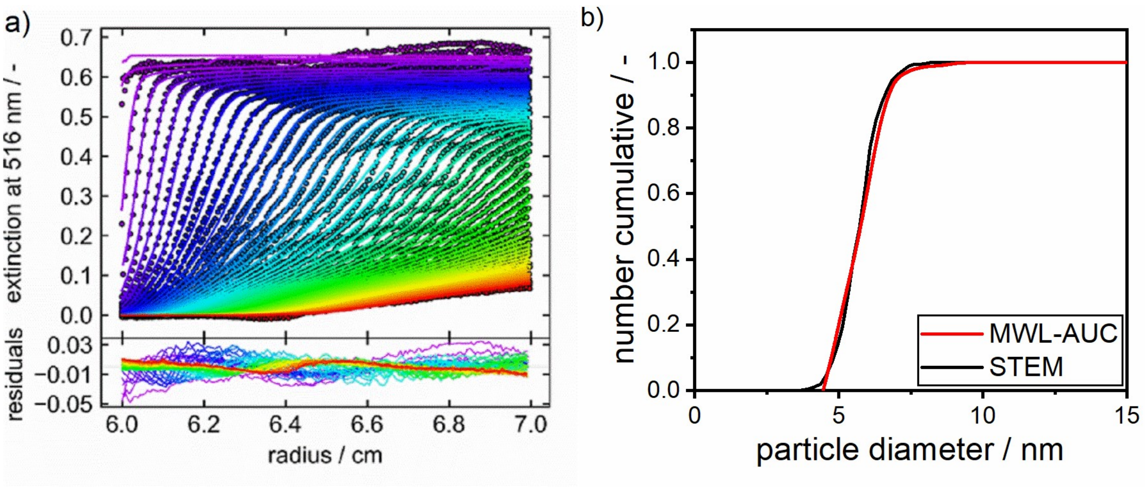

Figure S2: a) GUSSI plot of the performed core-shell analysis for an exemplary sample of gold NPs. b) Auxiliary MWL-AUC measurement for an exemplary alloy sample with a molar gold content of 75 %.

2D distribution for additional compositions

As described in the main text of the manuscript, the produced particles' distribution in size and chemical composition can be analyzed using an optical back coupling method based on MWL-AUC data. Here we present additional 2D plots for a wider range of compositions.

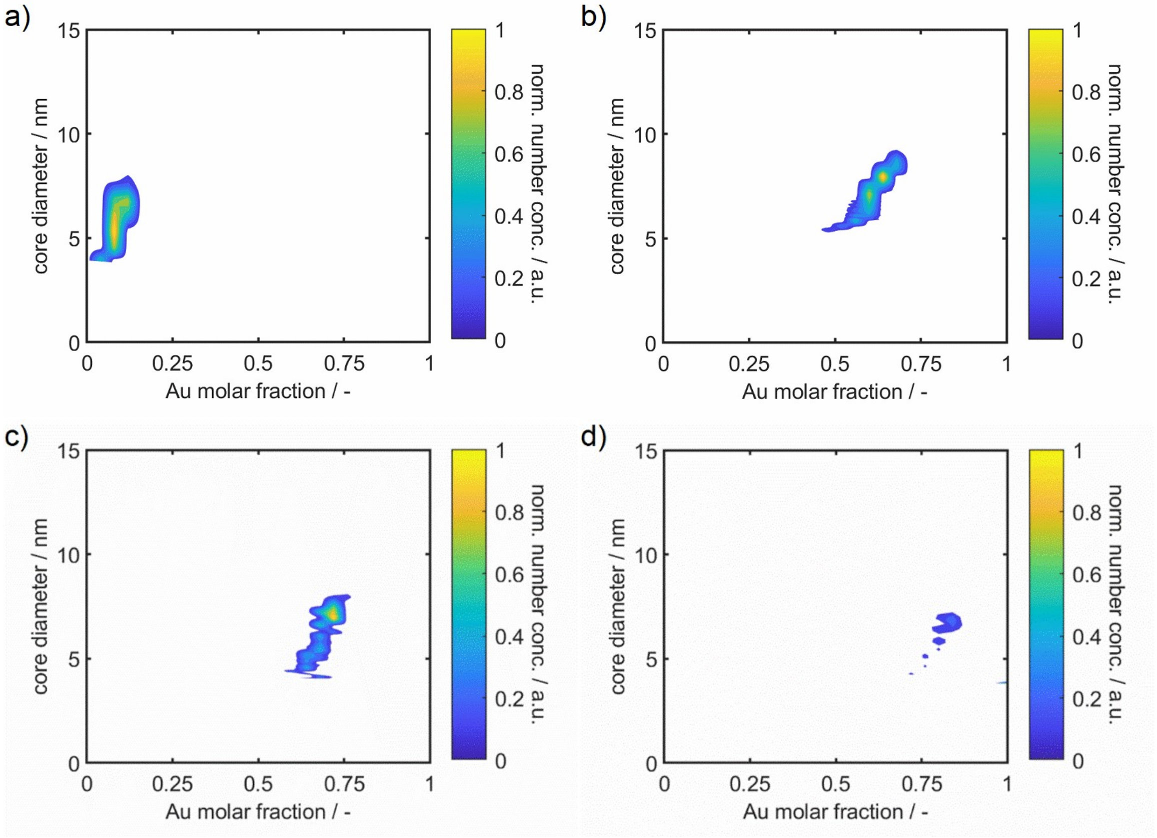

Figure S3: Two-dimensional property distributions of the synthesized alloy NP dispersion with a molar gold content of 10 % (a), 60 % (b), 70 % (c) and 80 % (d). The particle size is displayed on the y-axis and the gold content on the x-axis.

Remarkably, as mentioned in the main text, for silver-rich samples, a further ripening over multiple days has been detected (see Figure S4) during which the composition distribution narrowed itself. The reason for the artificial broadening of the composition distribution of the pre-ripened sample is a mismatch in the optical modelling as slight inhomogeneity results in a broadening of the extinction spectrum. This in turn is interpreted by the OBC algorithm as pronounced polydispersity in composition. For details it is referred to the recent work of Cardenas et al..[10]

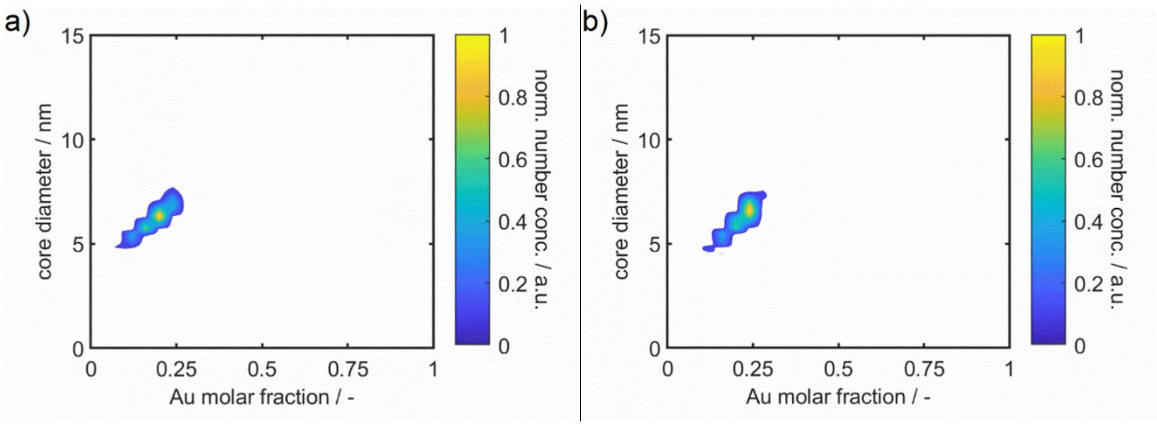

Figure S4: Two-dimensional property distributions of the synthesized alloy NP dispersion with a molar gold content of 25 %, 24 h after synthesis (a) and 72 h after synthesis (b).

Size Exclusion Chromatography

Size exclusion chromatography was performed to confirm mono-modality of the synthesized samples and determine colloidal stability. Two additional species with retention volumes of 6 mL and 12.5 mL apart from the alloy NPs were detected. Figure S5 b shows the spectra of the three identified peaks from low to high retention volumes.

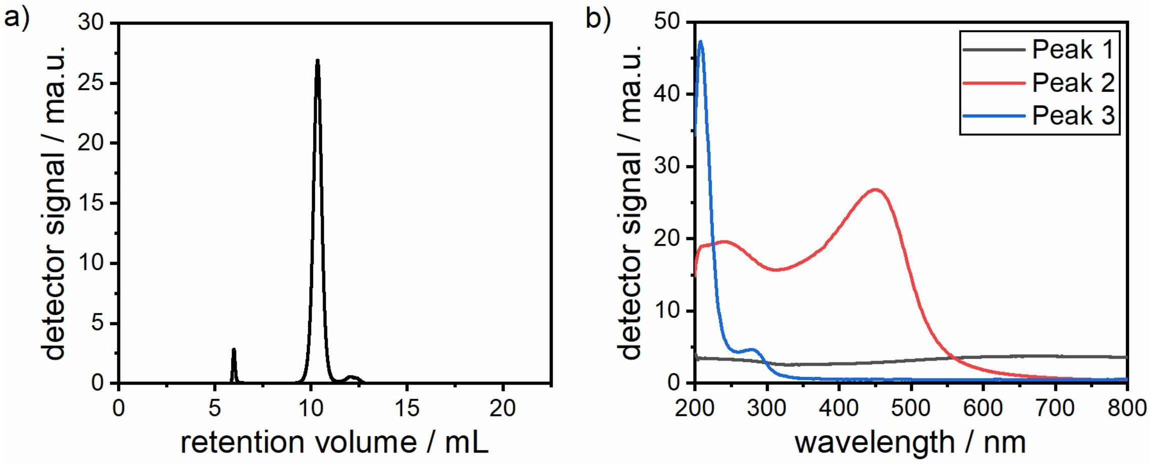

Figure S5: Chromatograms of alloy NPs with a molar gold content of 50 % (a) and the respective extinction spectra of the three detected peaks from low to high retention volumes (b).

Additional microscopy images

Scanning transmission electron microscopy images were taken via the GeminiSEM 500 from ZEISS equipped with a STEM detector. The particle size distribution was determined by manual measurement of the particles' diameter. Figure S 6 exemplarily shows an overview STEM image taken from a sample of silver-gold alloy NPs with molar gold content of 50 %.

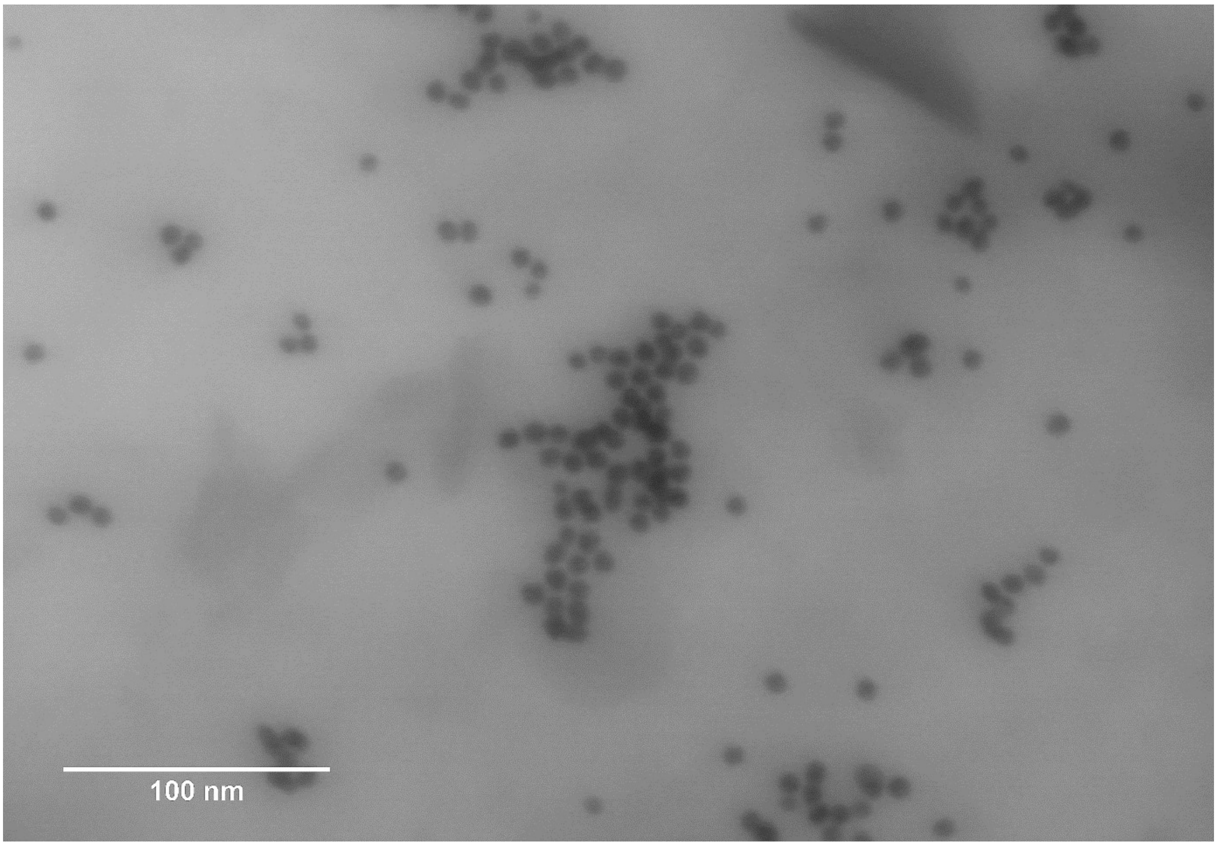

Figure S 6: STEM image of silver-gold alloy NPs with a molar gold content of 50 %. The image was taken via the GeminiSEM 500 from ZEISS equipped with a STEM detector.

Furthermore, we took HR-STEM images of the NPs to get a deeper insight into their morphology.

Figure S 7 shows HR-STEM images of silver-gold alloy NPs with a molar gold content of 0 to 100 % in 25 % intervals with the lattice fringes clearly visible.

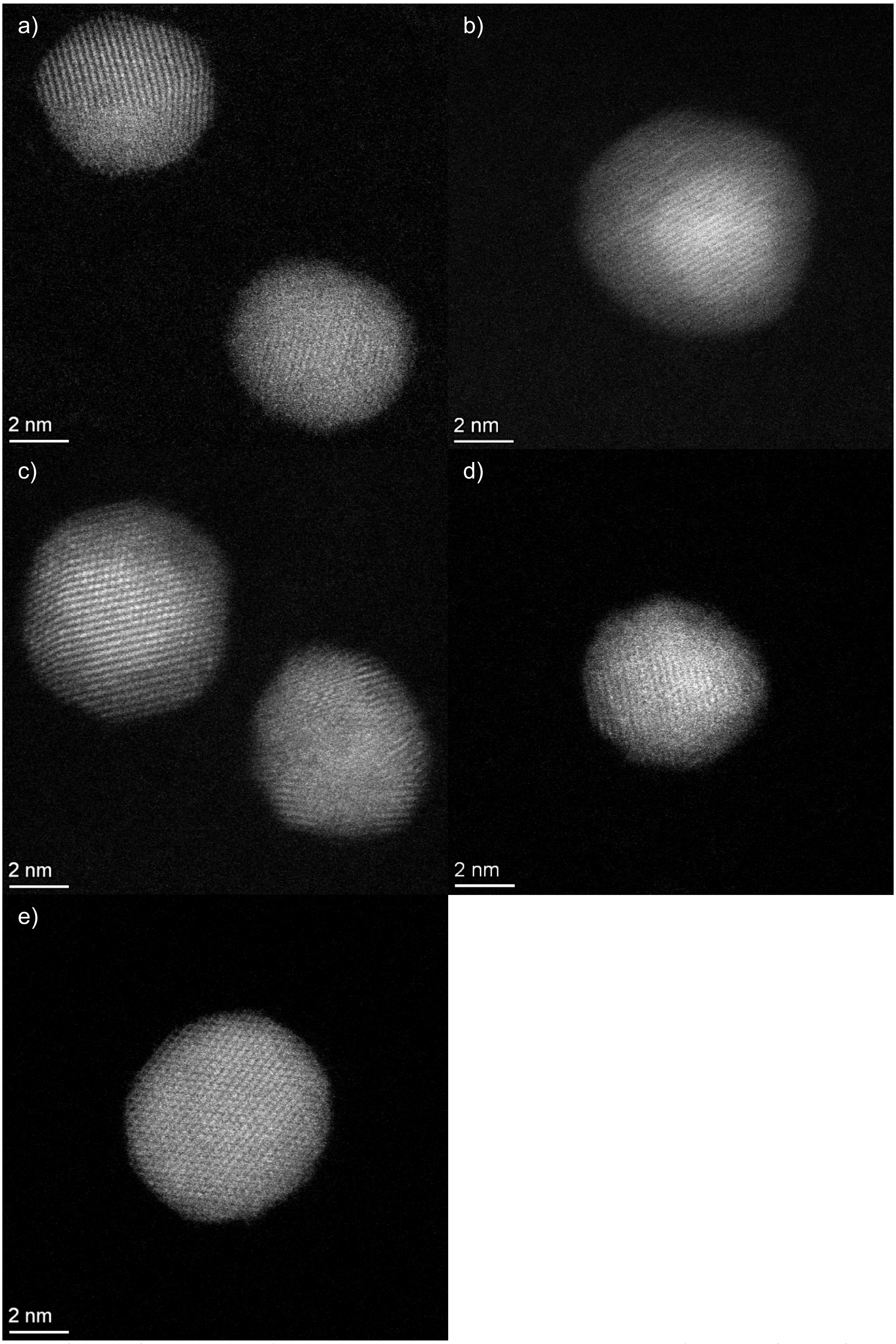

Figure S 7: HR-STEM images of silver-gold alloy NPs with a molar gold content of 0 to 100 % in 25 % intervals from a) to e) respectively. Images were taken via the FEI Titan Themis^{3} 300 TEM.

Particle size distributions

The particle size distributions, from which the cumulative distributions shown in the manuscript are derived, are shown as histograms. Additionally we included the Gaussian best fit. The data may be used for better comparability to literature data.

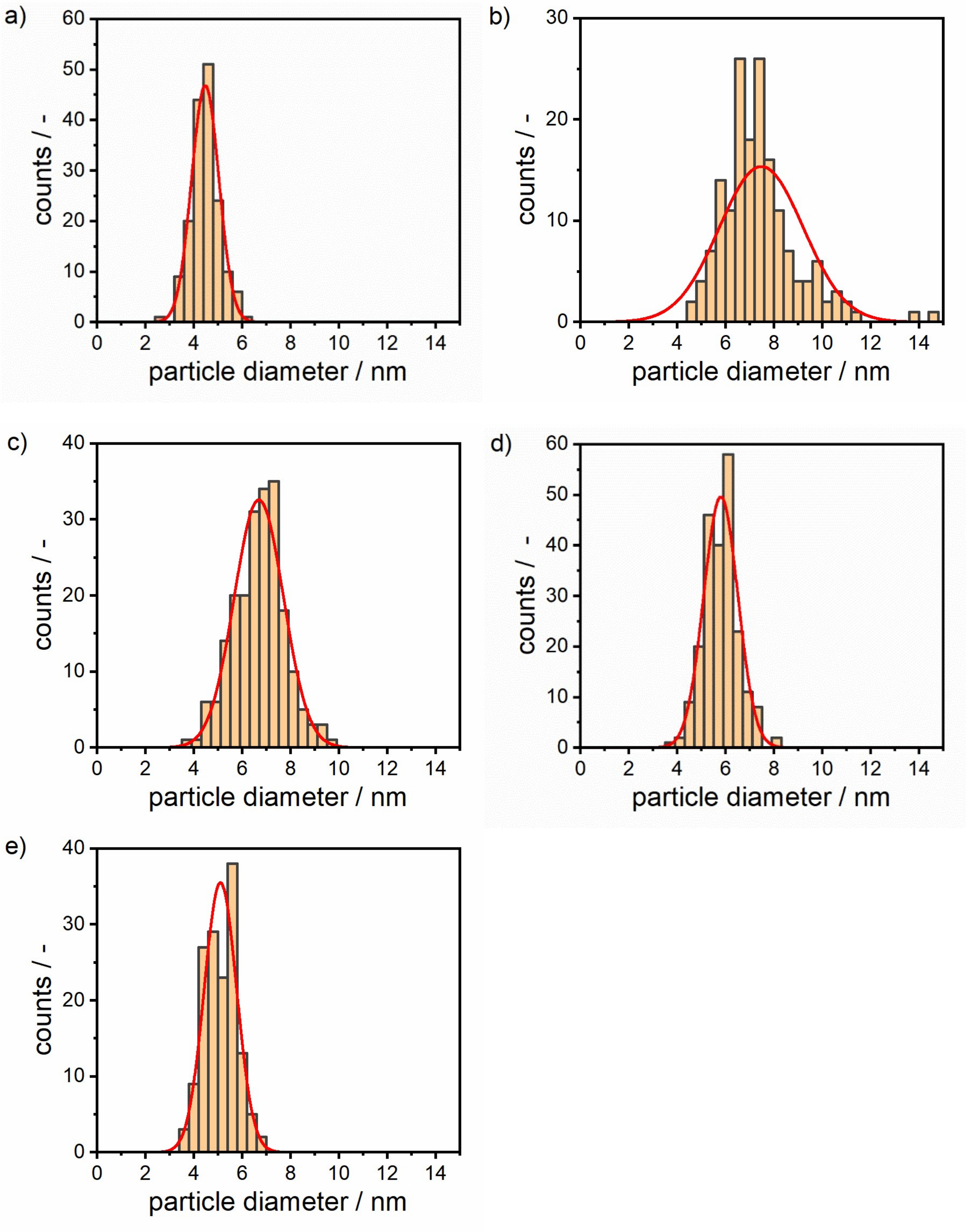

Figure S 8: Particle size distributions of particles with a molar gold content of 0 % to 100 % in 25 % increments from a) to e) respectively. All counts were obtained manually from STEM images.

Effective reaction rates

Effective reaction rates were determined from the change of the extinction value at the LSPR position of the respective particle system. We fit a linear equation to the region in the extinction-time diagrams, in which the extinction increases linearly with time. From the slope of this function, we then determine the effective reaction rate. Figure S 9 shows the process schematically for the formation kinetics of silver-gold alloy NPs with a molar gold content of 50 %.

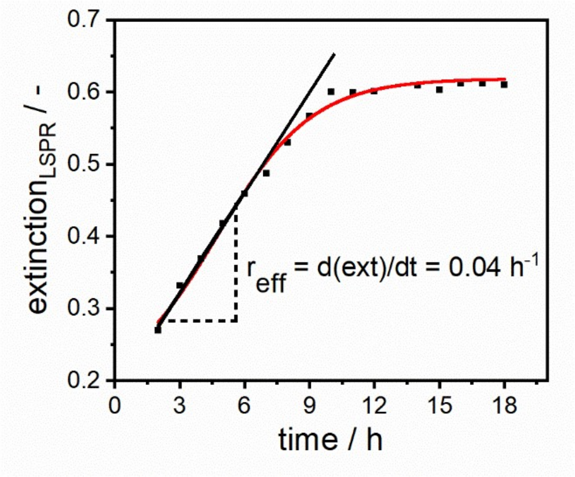

Figure S 9: Schematic determination of the effective formation rate of silver-gold alloy NPs with a molar gold content of 50 %.

Processibility of produced nanoparticles

For the industrial application of the NPs, their processibility is of major importance. While the stability of the particles in solution certainly is one factor to investigate, the system's behavior to drying and redispersion is another important aspect. Figure S 10 shows the extinction spectra as well as the chromatograms of an alloy sample before and after drying and redispersion. Drying was performed in a heating cabinet at 60 °C as well as in a freeze dryer, redispersion was realized by ultrasonication. For the particles dried at 60 °C a shift in the extinction spectrum towards higher wavelengths is observable. Additionally, a significantly increased amount of agglomerates is present, which can be seen by the increase in extinction at high wavelengths and by the increase and broadening of the agglomerate peak in the chromatogram at a retention volume of around 6 mL. The freeze-dried sample, however, shows no significant change in the optical properties after redispersion in water. While the extinction spectrum shows slightly increased values, which can likely be traced back to slight deviations in the added water after the drying process, the chromatogram shows a significant shift to higher retention volumes. This either indicates a strong interaction between the particles and the stationary phase material of the HPLC column or a decrease in the hydrodynamic diameter of the particles.

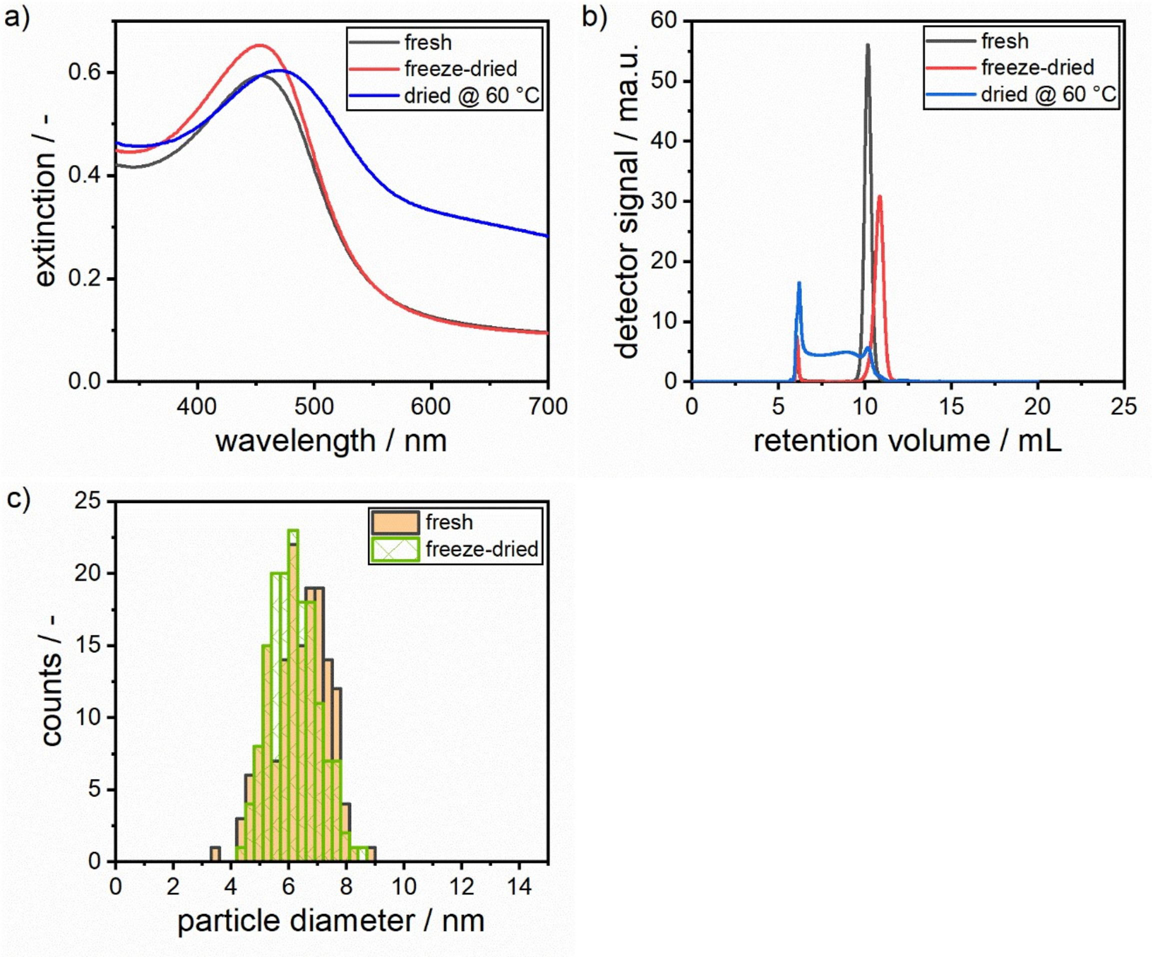

Figure S 10: a) Extinction spectra of produced alloy NPs with a molar gold content of 50 % before (fresh) and after their redispersion after a drying step. Drying was performed at 60 °C in a drying cabinet, as well as in a freeze dryer. b) Respective chromatograms of the 3 samples. c) Particle size distributions of the respective sample before (fresh) and after redispersion after a freeze-drying step.

The zeta potential of the sample before and after the drying process was determined to -41 mV and -37 mV, respectively, which rules out a stronger interaction with the column material, which is also negatively charged. We therefore further determined the PSD of the sample after the drying process and compared the obtained core sizes with a freshly prepared sample (see Figure S 10c). It was found that the core sizes of both samples match almost perfectly. Further investigations in this direction have not been performed. Overall, this proofs, that the particles produced via the dextran based synthesis introduced in this manuscript, can be freeze-dried, stored as a powder and redispersed later completely, which further increases its potential for industrial application.

References

1 W. Peukert, D. Segets, L. Pflug and G. Leugering, in *Mesoscale Modeling in Chemical Engineering Part I*, Elsevier, 2015, pp. 1–81.

2 G. Mie, Beiträge zur Optik trüber Medien, speziell kolloidaler Metallösungen, *Ann. Phys.*, 1908, **330**, 377–445.

3 J. Walter and W. Peukert, Dynamic range multiwavelength particle characterization using analytical ultracentrifugation, *Nanoscale*, 2016, **8**, 7484–7495.

4 Wiscombe and W. J, NCAR/TN-140+STR Mie Scattering Calculations: Advances in Technique and Fast, Vector-Speed Computer Codes.

5 J. Pearson, J. Walter, W. Peukert and H. Cölfen, Advanced Multiwavelength Detection in Analytical Ultracentrifugation, *Analytical chemistry*, 2018, **90**, 1280–1291.

6 The Stokes-Einstein law for diffusion in solution, *Proc. R. Soc. Lond. A*, 1924, **106**, 724–749.

7 P. Schuck, Size-Distribution Analysis of Macromolecules by Sedimentation Velocity Ultracentrifugation and Lamm Equation Modeling, *Biophysical journal*, 2000, **78**, 1606–1619.

8 P. Schuck and P. Rossmanith, Determination of the sedimentation coefficient distribution by least-squares boundary modeling, *Biopolymers*, 2000, **54**, 328–341.

9 J. Walter, G. Gorbet, T. Akdas, D. Segets, B. Demeler and W. Peukert, 2D analysis of polydisperse core-shell nanoparticles using analytical ultracentrifugation, *The Analyst*, 2016, **142**, 206–217.

10 P. Cardenas Lopez, M. J. Uttinger, N. E. Traoré, H. A. Khan, D. Drobek, B. Apeleo Zubiri, E. Spiecker, L. Pflug, W. Peukert and J. Walter, Multidimensional characterization of noble metal alloy nanoparticles by multiwavelength analytical ultracentrifugation, *Nanoscale*, 2022. DOI: 10.1039/D2NR02633C.
